# Risk stratification in transthyretin-related cardiac amyloidosis

**DOI:** 10.3389/fcvm.2023.1151803

**Published:** 2023-03-21

**Authors:** Riccardo Scirpa, Edoardo Cittadini, Lorenzo Mazzocchi, Giacomo Tini, Matteo Sclafani, Domitilla Russo, Andrea Imperatrice, Alessandro Tropea, Camillo Autore, Beatrice Musumeci

**Affiliations:** ^1^ Division of Cardiology, Department of Clinical and Molecular Medicine, Sapienza University of Rome, Rome, Italy; ^2^ Department of Cardiology, IRCCS San Raffaele Pisana, Rome, Italy; ^3^ San Raffaele Cassino (FR), Cassino, Italy

**Keywords:** risk stratification, cardiac amyloidosis, natural history, transthyretin, heart failure, arrhythmias

## Abstract

Transthyretin related cardiac amyloidosis (TTR-CA) is an infiltrative cardiomyopathy that cause heart failure with preserved ejection fraction, mainly in aging people. Due to the introduction of a non invasive diagnostic algorithm, this disease, previously considered to be rare, is increasingly recognized. The natural history of TTR-CA includes two different stages: a presymptomatic and a symptomatic stage. Due to the availability of new disease-modifying therapies, the need to reach a diagnosis in the first stage has become impelling. While in variant TTR-CA an early identification of the disease may be obtained with a genetic screening in proband's relatives, in the wild-type form it represents a challenging issue. Once the diagnosis has been made, in order to identifying patients with a higher risk of cardiovascular events and death it is necessary to focus on risk stratification. Two prognostic scores have been proposed both based on biomarkers and laboratory findings. However, a multiparametric approach combining information from electrocardiogram, echocardiogram, cardiopulmonary exercise test and cardiac magnetic resonance may be warranted for a more comprehensive risk prediction. In this review, we aim at evaluating a step by step risk stratification, providing a clinical diagnostic and prognostic approach for the management of patients with TTR-CA.

## Introduction

Transthyretin related cardiac amyloidosis (TTR-CA) is an infiltrative cardiomyopathy caused by extracellular deposition of transthyretin (TTR)-derived insoluble amyloid fibrils in the myocardium. TTR-CA is generally considered to be rare but in the last 20 years, due to advanced technology and improvement of diagnostic tools, it has been increasingly recognized (1). Two distinct types of the TTR protein (variant, vTTR, or wild type, wtTTR) become unstable, and misfolding forms aggregate resulting in amyloid fibrils.

The true prevalence of both forms of TTR-CA is hard to define, since the familial form present a highly uneven geographical distribution and the senile form is often underdiagnosed (2). The development of effective and specific drugs for TTR-CA marked the beginning of a new era for this disease once deemed incurable (3, 4).

**Figure 1:**
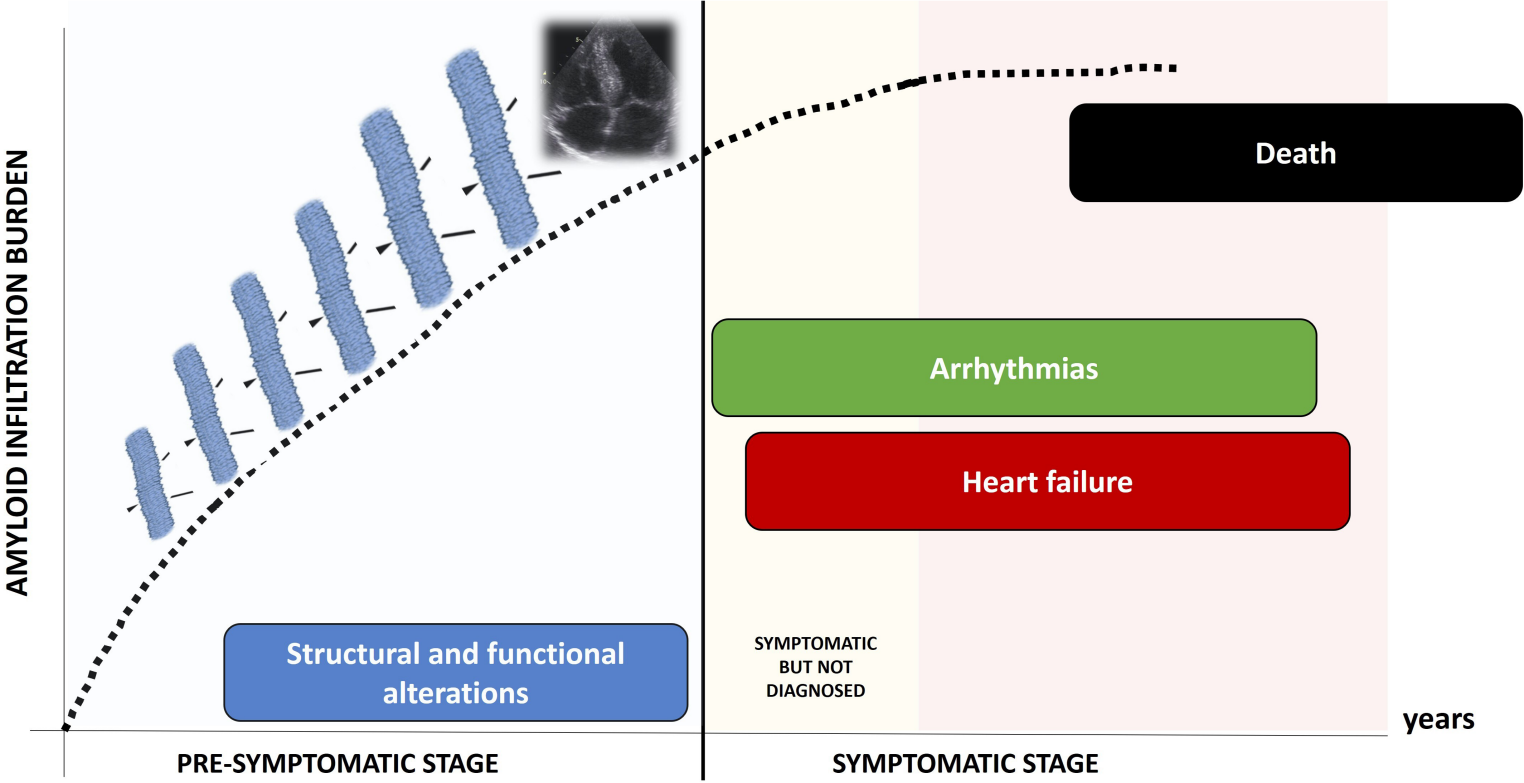
Natural history of TTR-CA: the pre-symptomatic stage and the symptomatic stage.

Clinical course of TTR-CA is characterized by two different stages (Figure 1): a *pre-symptomatic stage*, when fibrils progressively infiltrate the heart, causing initial and subclinical structural and functional alterations; in this stage, patients are usually asymptomatic and often elude the diagnosis; in a subsequent time, the *symptomatic stage*, the disease clinically manifests: patients suffer from progressive heart failure (HF), arrhythmias and conduction system disease, undergo clinical evaluation and are eventually diagnosed. Death occurs in a median time of 3–4 years (1).

To improve care and risk stratification of TTR-CA patients, the identification of clinical and instrumental features associated with both the development and the progression of the disease is of paramount importance. Although the number of diagnoses of TTR-CA has increased markedly during the last 20 years (5), the main challenge in the management of this disease still remains its early recognition. Indeed, most of patients achieve a diagnosis when they have already reached hard endpoints, as arrhythmias or HF hospitalizations. Moreover, the specific TTR-CA therapies result more effective in patients with mild symptoms, further emphasizing the need to identify TTR-CA patients before clinical conditions worsen and HF develops and progresses.

### Pre-symptomatic stage

Before the occurrence of clinical manifestations, clinical efforts need to be focused on recognizing those patients who can develop the disease and need to be screened.

In vTTR, more than 140 different mutations of the TTR gene have been described and specific variants often correlate with different clinical manifestations, ranging from a prevalent cardiac phenotype to mixed and prevalent neurological ones (6). The various mutations are associated with specific phenotypes and some of them are endemic in specific geographical areas (7). For example, the variant Val30Met has different manifestations and penetrance according to the geographical location, ranging from a fast-progressing disease dominated by neuropathy with early onset and high penetrance (Portuguese form), to a slowly progressing disease with late onset and low penetrance (Swedish form) (8, 9). Typical “cardiogenic” variants, like Val122Ile in North America and Ile68Leu in Italy, are characterized by clinical manifestations very similar to those of wtTTR, including a high prevalence of carpal tunnel syndrome (CTS), with a comparable age of onset (seventh-eight decade of life) (10, 11). These cardiac forms share with the wtTTR also the male prevalence, in contrast to mixed forms like those caused by Phe64Leu or Glu89Gln, in which the gender disparity is milder or absent (12, 13). In general, women who carry a pathogenic variant less likely have cardiac involvement and among asymptomatic carriers there is a relatively larger female presence (14), suggesting a lower penetrance in women. Furthermore, it has been reported that inheriting the pathogenetic TTR variant from the mother can cause an anticipation of disease onset and consequently a higher penetrance (15). In summary, carrying a pathogenic TTR mutation confers a variable risk of developing CA, which depends on the specific variant, the geographical area, gender and transmitting parent (father vs. mother). Genotype-positive phenotype-negative individuals should be periodically visited in order to detect the development of minor disease signs and in this way allow an early initiation of a specific therapy. It has been proposed that the clinical follow-up should start about 10 years before the predicted age of disease onset (PADO), estimated from the typical age of onset associated with the specific mutation, the age of onset of the affected relatives and the sex of the transmitting parent (16).

In wtTTR, extracardiac manifestations like CTS, which can represent an early sign of the systemic disease, are likely the only predictive factors of its development. CTS is a very frequent finding among TTR-CA patients, especially in the wild-type form, because of selective amyloid deposition in the transverse carpal ligament (17). This particular localisation may be explained by the presence of repetitive mechanical stimuli in carpal tunnel area, as well as in the heart, that facilitate TTR amyloidogenesis through the activation of plasminogen (18). Compared to the general population, the prevalence of CTS in TTR-CA is higher, ranging from 15% to 60%, especially in men in the seventh and eight decades (19, 20). It is well known that the diagnosis of CTS is often followed by the development of CA with a characteristic latency of 5–10 years (21). This interval is the most likely explanation for the low incidence of CA (2%) found by Sperry et al. (22) in patients undergoing carpal tunnel surgical release, despite the fact that amyloid deposits have been found in 10% patients. On the other hand, this approach offers the opportunity of a very early screening of patients at risk to develop TTR-CA in the following years. Indeed, a history of CTS has been associated with a 12 times higher risk of amyloidosis as compared to matched control subject without CTS; an odd that raises to 30 times in the case of bilateral CTS (23). A recent study (24) has shown that the prevalence of wtTTR, 5–15 years after surgery for bilateral CTS, reached 8.8% in men, getting closer to the aforementioned prevalence of TTR deposits in the carpal tunnel ligament (22), and suggesting that amyloid deposition in this specific site could predict future development of CA. Post hoc subgroup analysis has highlighted a prevalence of 25.7% in men >70 years old, after excluding patients with BMI > 30 and occupational risk factors for CTS. Moreover, this screening approach has allowed an early diagnosis, considering that almost all the TTR-CA patients identified had low disease severity scores (24). The presence of left ventricular (LV) hypertrophy or other red flags, especially NT-proBNP and a relative apical sparring pattern, may allow to increase the sensitivity of the screening method (25, 26).

Finally, some echocardiographic features may raise the suspicion of TTR-CA in the context of the LV hypertrophy. In a multicentric study, evaluating more than 1,000 patients with increased heart wall thickness, in which amyloidosis was suspected, relative wall thickness, evidence of diastolic dysfunction (E/e'), TAPSE and strain variables assessing the relative apical sparing had best diagnostic accuracy to individuate those with amyloid infiltration (27). Moreover, a simple score, obtained by the product of relative wall thickness and E/e' ratio, has been demonstrated to possibly have a role as an initial screening tool for patients with suspected TTR-CA (28). Recently, Merlo et al. in a multicentric Italian study enrolling 5,315 unselected consecutive patients undergoing echocardiogram for reasons other than known or suspected cardiac amyloidosis (CA), showed that 1.2% of them reached a diagnosis of TTR-CA. Echocardiographic findings as non-dilated, hypertrophic hearts with LV ejection fraction >50% in combination with apical sparing or at least two red flags (i.e., restrictive filling pattern, granular sparkling, pericardial effusion, interatrial septum thickness >5 mm, atrio-ventricular valve thickness >5 mm) provide a diagnostic accuracy >70% (29). An ECG discordance with echocardiographic findings of hypertrophied not dilated LV or a slightly increase of cardiac biomarkers further increase the suspicion of TTR-CA (1, 8).

### Symptomatic stage

#### Risk factors for heart failure and death

HF is the main complication of TTR-CA, both in terms of number of hospitalizations and of mortality. In recent years, TTR-CA has been increasingly recognized as a cause of HF hospitalizations, exceeding the rate of 65 cases per 100,000 people/year in several regions of the United States (30). Current pharmacological management of HF is limited in patients with TTR-CA: drugs as beta blockers and renin angiotensin system inhibitors may be poorly tolerated (31). Moreover, the reduced size of LV cavity and the frequent involvement of right ventricle may hamper the use of long-term ventricular assist devices. Finally, heart transplantation may be an effective option, but only in carefully selected patients (32).

**Figure 2:**
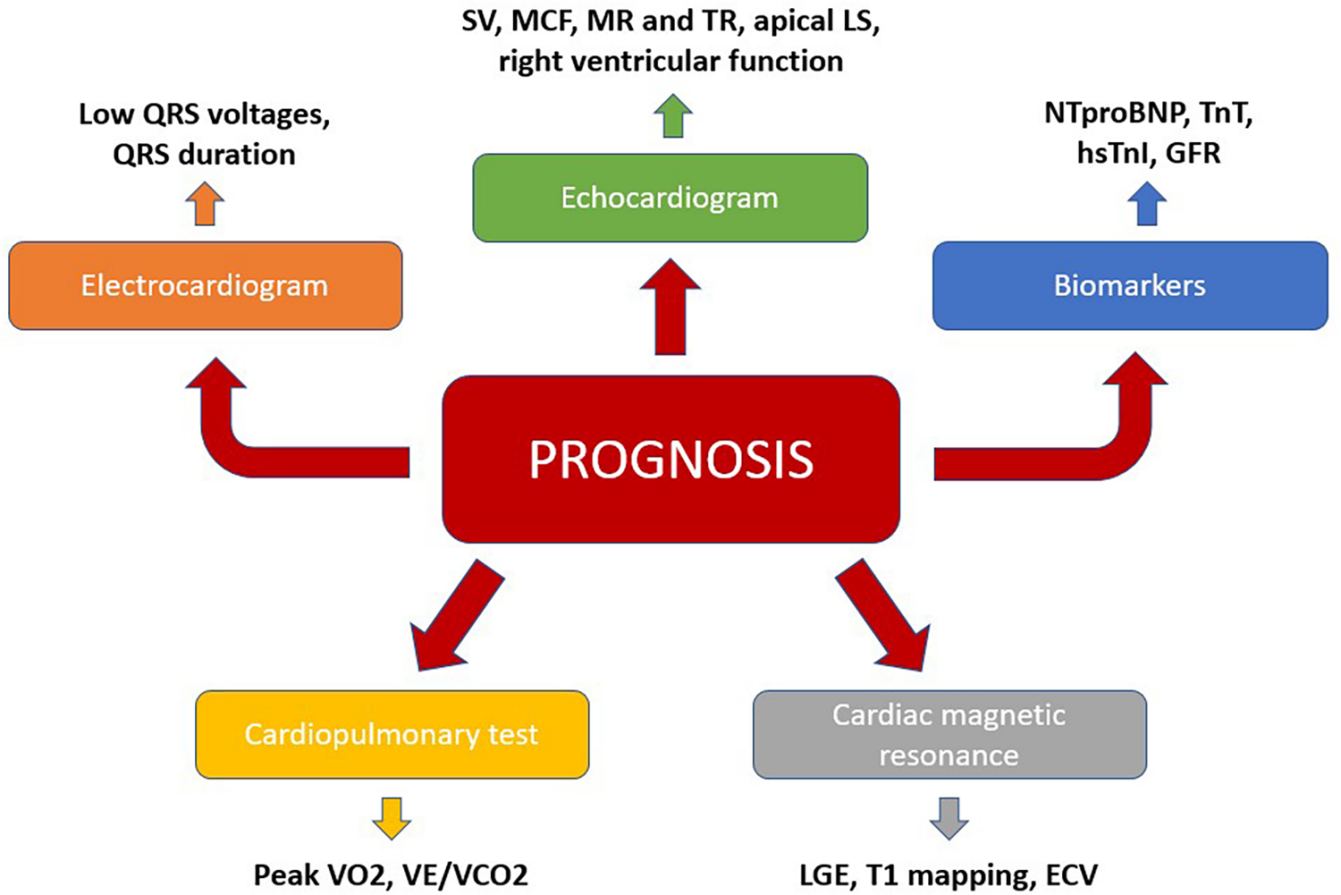
A multiparametric approach to predict prognosis in TTR-CA. ECV, extracellular volume; GLS, global longitudinal strain; GFR, glomerular filtration rate; LGE, late gadolinium enhancement; MR, mitral regurgitation; TR, tricuspid regurgitation; VCO_2_, carbon dioxide output; VE, minute ventilation; VO_2_, maximum oxygen consumption.

For these reasons, risk stratification of TTR-CA patients is imperative in order to identify patients with at risk of a faster disease progression towards HF, as this subset requires earlier and more aggressive therapies, as well as closer monitoring. In this regard, a multimodal approach, which integrates clinical, biomarkers and instrumental indicators need to be used (Figure 2).

##### Risk scores

In 2016, Grogan et al. (33) proposed for wtTTR a three-group classification system (Mayo score) according to cut-offs of troponin T (50 ng/L) and NT-proBNP (3,000 ng/L). The 4-year survival was 57%, 42% and 18% for stage I (both values below the cut-off), stage II (one marker above the cut-off) and stage III (both markers above the cut-off), respectively. This staging system is not validated for vTTR. Moreover, the use of troponin T appeared to be overwhelmed by the current worldwide diffusion of newer high sensitivity troponin assays, which not only differ in sensitivity, but also give different numerical results as compared to older troponin assays. In 2018, Gillmore et al. (34) developed a three-stage grading system (NAC score) for vTTR and wtTTR amyloidosis using NT-proBNP (cut-off: 3,000 ng/L) and glomerular renal filtrate (cut-off: 45 ml/min/1.73 m^2^) with median survival of 69, 47 and 24 months in stage I, II and III, respectively. Since the number of early diagnosis has recently increased, a very early stage (Ia) defined by low NT-proBNP concentration (<500 pg/ml, < 1,000 pg/ml with atrial fibrillation) and need for low doses of loop diuretics (<0.75 mg/kg) has been proposed (35) to better risk stratify CA patients. Stage Ia patients had a longer median survival compared with stage Ib (>100 months vs. 75 months), comparable to the age- and gender-matched general population. Despite this, a considerable cardiovascular morbidity still characterizes this stage, getting worse during the follow-up period, even in the patients with primary non-cardiovascular clinical presentation (36). Besides the aforementioned inter-assay variability of troponin, the accuracy of the “Mayo” staging system (33) is limited by the incompleteness of data regarding some variables and by the lack of external validation. Compared with NAC staging system, externally validated in an unrelated French cohort (34), it provides less effective prognostic discrimination, especially between stage I and stage II (35).

Alongside these two scores, other clinical markers have been proposed in recent years to improve their accuracy. In 2020, Cheng et al. (37) demonstrated that diuretic dose and NYHA functional class were strong independent predictors of all-cause mortality and the composite outcome of all-cause mortality or cardiac transplantation. This study, including both vTTR e wtTTRpatients, reported the incremental value of these parameters added to the Mayo and NAC staging systems. According to a recent study by the University of Heidelberg (38), the risk score proposed by Gillmore et al. for TTR-CA may not be sufficient to predict outcomes leading to advanced HF. In this study, a simple risk stratification score (“HeiRisk” score) including clinical parameters and biomarkers was generated to identify patients with end-stage cardiac light-chain (AL) or TTR amyloidosis in order to facilitate clinical decisions, such as listing for heart transplantation. This study showed that only cardiac biomarkers - hsTnT (cut off: 55 pg/ml) and NT-proBNP (cut off: 6,330 ng/L) - and QRS duration (cut off: 104 ms), but not haemodynamic measures, were significant predictors in TTR-CA.

However, all these scores are binary systems with few variables and this, whilst ensures simplicity of use, may be a limitation for an accurate prediction of outcomes, essential to evaluate the effects of new therapies; for this purpose a multiparametric approach is probably required.

##### Electrocardiogram

The most striking electrocardiographic abnormality in patients with CA is the reduction of QRS voltages, particularly in the limb leads, and the disproportion between QRS voltages and LV thickness at echocardiography (39). Low QRS voltage is defined as a QRS amplitude <5 mm (0.5 mV) in all peripheral leads. This finding, considered pathognomonic of the disease, is present in 60% of AL and only in 20% of TTR-CA, and may reflect the burden of amyloid infiltration in the heart. In a recent study by Cipriani et al. (40), low QRS voltages paired with an advanced disease stage independently predicted cardiovascular death. Together with the NAC staging, low QRS voltages provided incremental prognostic value in TTR-CA.

##### Echocardiogram

Cardiac amyloid deposition usually causes HF with preserved ejection fraction. Therefore, different echocardiographic tools have been proved useful to define the prognosis of TTR-CA patients, beyond LV ejection fraction. A decreased SV index, which represents a marker of advanced disease, has shown to independently predict prognosis, even after adjustment for NYHA class and NAC staging system (41). Chacko et al. have demonstrated a progressive worsening of structural and functional echocardiographic parameters over time, although only worsening in the degree of mitral and tricuspid regurgitation at 12- and 24-month assessments associated with a worse prognosis (42). Moreover, myocardial contraction fraction (MCF), which is the ratio of LV systolic output to LV myocardial volume, has shown promising result to predict outcomes in CA patients. In the THAOS registry (43) the median survival of patients with MCF < 25% was less than 3 years compared with 6.8 years of patients with MCF ≥ 25%.

In recent years, assessment of LV global longitudinal strain (GLS) has proved to be of great diagnostic and prognostic significance. In patients with TTR-CA, GLS is reduced showing the characteristic apical sparing pattern with a “cherry on top” appearance at the bull's eye plot. Recently, a reduction in apical longitudinal strain (cut off: −14,5%) have shown to be an independent predictor of major cardiac adverse events (44). On the contrary, longitudinal strain of the basal and midcavity sections, where amyloid infiltration is more marked and early, has not been found to predict prognosis (44). These data suggest that a reduction of apical longitudinal strain, typical of the advanced stages of the disease with severe amyloid deposition, is uncommon and probably less helpful in the early course of CA.

Right ventricular dysfunction, assessed by TAPSE (cut off: 14 mm), has been associated with a higher rate of cardiovascular events (45, 46). A recent study (46) has also highlighted that right ventricular free wall strain (cut off: 16%) may have an independent prognostic role for all causes of death. A study by Bandera et al. (47) demonstrated that increased atrial stiffness, identified using echo speckle tracking and characterized by a reduction in the reservoir and contractile function of the atrium, remains independently associated with prognosis after adjusting for known predictors. Notably, the absence of atrial contraction,foundin 22% of patients in sinus rhythmis associated with a significantly poorer prognosis compared topatients who maintain an effective mechanical contraction,andsimilar tothose with atrial fibrillation (47).

##### Cardiopulmonary exercise test

The cardiopulmonary exercise test (CPET) is the gold standard test todetermine prognosis in chronic HF with reduced ejection fraction (48). CPET is performed to assess the cardiocirculatory exercise response, together with the ventilatory and peripheral muscular responses. All of these parameters can be altered in amyloidosis due to the restrictive cardiomyopathy, cardiac denervation and chronotropic insufficiency. The main CPET characteristics of CA patients include reduced peak VO_2_, increased VE-VCO_2_ slope and episodes of oscillatory ventilation (EOV) (49). Peak VO_2_ and circulatory power has been found to be strongly and independently predictive of death or HF (50, 51). The combination of peak VO2 (cut off: 13 ml/min/kg) and NT-proBNP was the best predictor of all-cause mortality and the composite of mortality or HF-related hospitalization (45). Furthermore, the increase in VE/VCO_2_ slope (cut off: 40), resulted from several factors like autonomic dysfunction, right ventricular dysfunction and the absence of tidal volume rise during exercise, was shown to be associated with clinical events in wtTTR (49, 52).

##### Cardiac magnetic resonance

Cardiac magnetic resonance (CMR)has the ability to provide unique information about myocardial tissue composition. Indeed, it can identify and quantify cardiac amyloid deposition, using late gadolinium enhancement (LGE) and T1 mapping with calculation of extracellular volume (ECV).

In CA, LGE shows a characteristic global subendocardial pattern, generally associated with abnormal myocardial and blood-pool gadolinium kinetics (53). Non-contrast T1-mapping has great diagnostic accuracy for CA, being more sensitive than LGE imaging for identifying early disease (54). Transmural LGE has been associated with higher mortality compared to subendocardial pattern, remaining an independent negative predictor of survival in multivariable Cox models, as well as NT-proBNP and stroke volume indexed (55). Both native T1 mapping and ECV correlate with mortality, but only ECV remains independently predictive of prognosis after adjustment for other prognostic factors, as evidence of its robustness as a marker of cardiac infiltration (56).

### Risk factors for arrhythmias

Although the clinical course of TTR-CA is dominated by HF and its manifestations, arrhythmias and conduction system diseases are also very common (57). Sudden cardiac death has been reported to be one of the main causes of death (58), although often from pulseless electrical activity. Moreover, cardiac arrhythmias are associated with increased in-hospital mortality and acute HF exacerbations (59).

Atrial fibrillation is the most commonly observed heart rhythm disturbance in CA, especially in wtTTR, where it can be detected in up to 70% of patients (60, 61). The progressive diastolic dysfunction and the increase of filling pressures, together with the selective deposition of amyloid in the atria walls (62), lead to atrial structural and functional remodeling - also called atrial myopathy -, which accounts for the frequency of supraventricular arrhythmias. Age, HF, LV ejection fraction, left atrial size and right atrial pressure have shown to be independent predictors of developing atrial fibrillation (63). A history of atrial fibrillation is strongly associated with prevalent and incident HF (63); however, in contrast to other etiologies of HF, in TTR-CA atrial fibrillation doesn't seem to impact survival and all-cause mortality (60, 61, 63). Previous studies have emphasized the high prevalence of intracardiac thrombi in CA, in particular in patients with atrial fibrillation (64, 65). Restrictive filling pattern and low left atrial appendage emptying velocities at transesophageal echocardiogram have been shown to predict the presence of intracardiac thrombi (63). Furthermore, a significant proportion of arterial thromboembolic events occurred in patient in sinus rhythm or despite adequate anticoagulation therapy due to the amyloidosis related atrial myopathy, that causes a progressive decline of atrial function and, eventually, an electromechanical dissociation (66, 67). In view of this, in patients at high risk of thromboembolic events the execution of a transesophageal echocardiography should be considered before direct current cardioversion (68, 69).

High-grade atrioventricular (AV) blocks are present in 9.5% of TTR-CA patients at the time of diagnosis (70). Amyloid fibrils infiltrate the conduction system, making an increasing number of patients pacemaker (PMK)-dependent as the disease progresses (71). Several studies reported that device implantation is required in about 9%–11% of patients in the years following the diagnosis (70–72). PMK implantation impacts on outcomes, as right ventricular pacing may be associated with worsening HF symptoms, LV ejection fraction decline and mitral regurgitation severity (73). In a recent paper, it has been showed that history of atrial fibrillation, PR interval >200 ms and QRS duration predict future PMK implantation. The presence of these features should advice a close monitoring, while the absence of all these risk factors allow to exclude with great accuracy the need of PMK in the first 6 months after diagnosis (72).

Ventricular tachyarrhythmias, although frequent, have not been thought to contribute significantly to overall mortality in CA, especially TTR-CA (74). On the other hand, previous studies (75, 76) have reported a high rate of appropriate and successful implantable cardioverter defibrillator (ICD) therapies, even if involving mostly AL patients. A recent retrospective study cohort of 130 TTR-CA patients (77) have documented a high rate of ventricular arrhythmias and appropriate ICD therapies, in particular in those patients with systolic dysfunction. The evidence of non-sustained ventricular tachycardia (NSVT) and a history of unexplained syncope has been proposed as criteria for ICD implantation (78). In contrast, in a recent meta-analysis, the predictive value of NSVT has been debated and it has been shown that a NYHA class III-IV is associated with lower rate of appropriate ICD therapies. The physiopathological explanation of this result is that the focal amyloid deposits and associated fibrosis in the early stage of the disease can act as arrhythmogenic foci (79).

However, no studies have demonstrated a survival benefit related to ICD implantation, highlighting the need to better select patients at risk of lethal arrhythmic events. Furthermore, in an elegant study (80) CA was associated with a mortality rate of 26.9% at 1 year after ICD implantation compared with 11.3% among a propensity-matched cohort of patients with other non-ischemic cardiomyopathies; in this context the Authors found 5 predictors of mortality: a history of syncope, NSVT, diabetes mellitus, cerebrovascular disease and renal dysfunction. Therefore, it is clear that the risk of lethal arrhythmias should be balanced with the risk of other competitive causes of mortality. In this regard, ICD implantation should probably be considered in patients with lesser cardiac involvement and in the early stages of the disease (81).

### Therapeutic implication of disease staging

The main goal of the emerging disease-modifying therapies - TTR gene silencers and TTR stabilizers - is to prevent further generation or deposition of amyloid fibrils. For this reason, an early diagnosis and a prompt start of this specific treatment allows to obtain a significant benefit in terms of survival and quality of life. On the other hand, patients with delayed diagnosis and advanced disease are unlikely going to benefit from these therapies (82). This is especially true in older patients with higher risk of competitive non-cardiovascular causes of mortality. Moreover, in the ATTR-ACT study (3) patients with NYHA class III disease at baseline had higher rates of cardiovascular-related hospitalizations, suggesting an unfavourable cost-benefit ratio of Tafamidis in this subgroup of patients. Risk scores have not been systematically used as criteria for inclusion or exclusion of patients in trials, neither as endpoints to determine drug efficacy. Nevertheless, it is reasonable to think that the use of these scores would be informative of the potential benefit of the treatment or of its futility.

## Conclusion

TTR-CA is increasingly recognized, particularly in older patients. The advent of new disease-modifiers therapies highlights the importance of reaching the diagnosis early, ideally in the pre-symptomatic stage. A multiparametric approach, including not only biomarker scores, but also clinic, electrocardiographic and imaging data, is suggested for a careful risk stratification of mortality and HF-related events, in order to tailor CA management and therapy and to improve outcomes. Identifying reliable predictors of arrhythmic events is still an unmet need and the role of ICD in CA remains unclear. The improvement of survival hopefully related to new therapies will likely change this scenario.
